# Application of New Radiosensitizer Based on Nano-Biotechnology in the Treatment of Glioma

**DOI:** 10.3389/fonc.2021.633827

**Published:** 2021-03-22

**Authors:** Yandong Xie, Yuhan Han, Xuefeng Zhang, Hongwei Ma, Linfeng Li, Rutong Yu, Hongmei Liu

**Affiliations:** ^1^ Institute of Nervous System Diseases, Xuzhou Medical University, Xuzhou, China; ^2^ Department of Neurosurgery, The Affiliated Hospital of Xuzhou Medical University, Xuzhou, China; ^3^ Department of Neurosurgery, The Affiliated Brain Hospital of Nanjing Medical University, Nanjing Medical University, Nanjing, China; ^4^ Department of Neurosurgery, Suqian First People’s Hospital, Suqian, China

**Keywords:** nano-radiosensitizer, radiotherapy, radiation sensitization, nanoparticles, glioma

## Abstract

Glioma is the most common intracranial malignant tumor, and its specific pathogenesis has been unclear, which has always been an unresolved clinical problem due to the limited therapeutic window of glioma. As we all know, surgical resection, chemotherapy, and radiotherapy are the main treatment methods for glioma. With the development of clinical trials and traditional treatment techniques, radiotherapy for glioma has increasingly exposed defects in the treatment effect. In order to improve the bottleneck of radiotherapy for glioma, people have done a lot of work; among this, nano-radiosensitizers have offered a novel and potential treatment method. Compared with conventional radiotherapy, nanotechnology can overcome the blood–brain barrier and improve the sensitivity of glioma to radiotherapy. This paper focuses on the research progress of nano-radiosensitizers in radiotherapy for glioma.

## Introduction

Glioma is a tumor originating from glial cells, which is the most common primary malignant tumor in the brain (1). According to the grade of malignancy listed in the National Comprehensive Cancer Network (NCCN) Guidelines Version 1.2020 Central Nervous System Cancers (CNS), gliomas are classified into grades I to IV. Grade I lesions are benign, including pilocytic astrocytoma, multiform yellow astrocytoma, ganglion glioma, and subependymal giant cell astrocytoma. Grade II tumors include diffuse astrocytomas and oligodendrogliomas, which grow slowly, but can be highly differentiated. However, differing from pilocytic astrocytomas, these tumors infiltrate normal brain tissue and have a tendency to turn malignant. Grade III tumors include anaplastic astrocytoma and oligodendroglioma, which are characterized by high cell density and mitotic cells. The tumors of Grade IV are the most damaged and most common gliomas, including glioblastoma and gliosarcoma. Although we have made many efforts in the past few decades, glioma still has not been cured, and the median survival time of glioblastoma is still only 12 to 15 months (2, 3). The prognosis for patients with recurrent disease remains poor, with a median survival of only 25 and 40 weeks for recurrent glioblastoma (GBM) and recurrent anaplastic glioma, respectively (4). Due to the active proliferation of glioma cells and the strong ability of invasive growth, the course of the disease progresses rapidly and is prone to recurrence and spread. As a routine treatment for glioma, radiotherapy has been used in clinical practice since 1970. The 2005 NCCN Glioma Treatment Guidelines recommend radiotherapy as one of glioma standard treatment methods (5–7).

Recently, radiotherapy has been developed rapidly, taking on an increasingly prominent role and position in the treatment of glioma, including conventional radiotherapy, three-dimensional conformal radiotherapy (3D-CRT), intensity-modulated radiation therapy (IMRT), and stereotactic radiotherapy. Conventional radiotherapy for gliomas mostly uses linear accelerators for whole-brain irradiation, which can easily cause damage to normal brain tissue and affect the radiotherapy dose in the tumor area. Radiotherapy technology has gradually shifted from whole-brain radiotherapy to local radiotherapy, together with improvements and research made when applying radiosensitizers, radiation doses, and radiation time intervals, in order to optimize the effect of radiotherapy, inhibit tumor progression, and improve radiation damage. However, radiotherapy for glioma still has some obvious shortcomings. For example, Roshan Karunamuni (8) found that radiotherapy for intracranial tumors can induce cognitive impairment, which is positively correlated with radiation dose. There was no significant difference in 5-year survival between patients with WHO grade II glioma (LGG) in the two groups who received 50.4Gy and 64.8Gy (9). NCCN recommends the use of preoperative and postoperative MRI imaging to determine the optimal tumor volume (GTV) and clinical target volume (CTV) before radiotherapy for gliomas. The clinical target volume (CTV) is an extension of the GTV (including Grade III gliomas, which increase the margin of 1 to 2 cm, and Grade IV gliomas, which increase the margin of 2 to 2.5 cm). Adult low-level glioma (WHO I or II) should receive 45–54Gy and 1.8v2.0Gy each time. For IDH wild-type low-grade glioma, increasing the RT dose to 59.4–60 Gy was considered. Anaplastic glioma and glioblastoma (WHO grade III or IV) recommend conformal RT (CRT) technology, including three-dimensional CRT (3D-CRT) and IMRT for focal brain irradiation, and the recommended radiation dose, with 60Gy and 2.0Gy each time or 59.4Gy and 1.8Gy each time. The initial radiotherapy plan was 46Gy and 2Gy each time.

The mechanism of radiotherapy is mainly divided into two types: direct damage and indirect damage. Direct damage is mainly caused by the direct action of radiation on organic molecules to produce free radicals to cause DNA molecules to break. Indirect damage is mainly caused by the ionization of water in human tissues by radiation (10). More and more studies have shown that the currently used low-liner energy transfer (Low-liner energy transfer LET) radiotherapy may promote the invasion and migration of gliomas (11). The radioresistance of gliomas is an important reason for the limitations of clinical radiotherapy. Rapid proliferation, high invasiveness, and radiation resistance are the main reasons behind unsatisfactory radiotherapy effects for gliomas. How to increase the radiosensitivity of glioma has become an important challenge (12–14).

The emergence of radiotherapy sensitizers provides new opportunities for radiotherapy for glioma. On the one hand, it can enhance the radiosensitivity of tumor cells; on the other hand, it can reduce the radiation dose and the adverse effects of normal brain tissue. When applied with radiotherapy, it can change the responsiveness of tumor cells to radiation, thereby improving the therapeutic efficiency. The killing effect of radiosensitizers on tumor cells is related to many factors, including tumor cell type, degree of cell differentiation, cell cycle, clinical stage, and anatomical classification (15). After treatment with radiation, DNA double-strand break (DSB) and DNA single-strand break (SSB) can be observed. Nevertheless, then some proteins related to DNA repair, such as DNA-dependent protein kinase (DNA-PK), and are activated to start the repair process. After that, the damaged cells return to normal cells eventually. In the process of radiation on cells, many factors determine the final results (16). Considering a single cell, it can enhance DNA damage and promote cell apoptosis or autophagy. Substances that inhibit DNA damage repair may enhance the killing effect of radiation on tumor cells to achieve the purpose of radiation sensitization. From the perspective of the tumor as a whole, the oxygen and state of the cells inside the tumor and the cell cycle distribution of the tumor cells have an impact on the killing effect of radiation. Most of the radiosensitizers used in the past refer to drugs with the abovementioned functions. With the continuous development of molecular biology, some small interfering RNA (siRNA) and monoclonal antibodies targeting radiation-sensitive genes have become new candidates for radiosensitizers (17, 18).

Adams and Fowler et al. divided traditional radiosensitizers into the following categories: DNA precursor base analogs (such as 5-BUdR), electrophilic radiosensitizers (including nitroimidazoles, nitroaromatic hydrocarbons, and nitro heterocyclic compounds), oxygen-like compounds, radiation damage repair inhibitors, mercapto inhibitors (such as 4-ethylmaleimide (NEM), neoarsphenamine, p-chloromercuribenzoate, iodoacetamide), cytotoxic compounds Sensitizer (Cu^2+^), tumor vascular disrupting agent, and gene-related tumor radiosensitizer, etc. (19, 20). At present, the conventional radiotherapy sensitizers in clinic include 5-fluorouracil, platinum (such as cisplatin, carboplatin), gemcitabine, etc., which can enhance the radiotherapy sensitivity of tumor cells through different mechanisms of action (such as inhibiting DNA synthesis, promoting DNA double-strand breaks, regulating the cell cycle, etc.) (21, 22). However, these conventional radiotherapy sensitizers also have some drawbacks. With the combination of radiotherapy to treat tumors, 5-fluorouracil has a short half-life and requires long-term intravenous drip administration, which easily forms thrombus and causes nosocomial infections (23). Cisplatin is a widely used clinical radiotherapy (CRT) drug, which can kill many types of tumors (24, 25). Consequently, it can cause many adverse reactions, such as nausea, vomiting, neurotoxicity, ototoxicity. and nephrotoxicity (26). 5-Iodine-2 deoxyuridine (IUdR) has been confirmed to have a significant radiosensitization effect on glioblastoma, but due to the short circulating half-life and the inability to pass the blood–brain barrier (BBB), its clinical application is limited (27). DNA double-strand repair inhibitors (DSBRIs) KU55933 were once considered as one of the most promising drugs to improve radiotherapy, but its clinical application remains due to its potential toxicity to normal tissues, inability to select-enter tumor cells, and poor solubilization (28). Misonidazole is a hypoxic cell sensitizer, which can enhance the antitumor effects of cyclophosphamide in preclinical studies (29). Formerly, it is expected to be an ideal radiotherapy sensitizer in terms of controlling radiation-resistant tumor cells and p53 mutant tumor cells (30). However, researchers in a randomized study found that Misonidazole did not improve the prognosis of cervical cancer radiotherapy compared with the placebo group (31), making people question the effectiveness of Misonidazole, with the toxicity of Misonidazol further studied. Trans sodium crocetinate (TSC) has been verified as a radiotherapy sensitizer. In a study of a C6 glioma model, the use of TSC improved the regression of GBM tumors after radiotherapy, increased survival, and achieved radiosensitization. The mechanism of action may temporarily increase tissue oxygenation of hypoxic glioma (32, 33). However, the effect on patients with glioma needs to be further explored. Carbon ion radiotherapy is an excellent way of radiotherapy, with great application prospects in glioma (34–36). However, its combination with nano-radiosensitizers remains to be studied.

Therefore, how to find a safe and effective radiotherapy sensitizer for glioma has become an urgent problem. With the rapid development of nano-science and technology, people are paying more and more attention to the role of nano radiation sensitizers in the treatment of glioma. Therefore, this paper will review the principle and types of radiosensitizers in radiotherapy for glioma and the research progress of radiosensitizers in radiotherapy for glioma

## Advantages of Nano-radiosensitizers in Radiotherapy for Glioma

Nanomaterials have been widely used to improve the efficacy of radiotherapy due to their good biocompatibility, inherent radiosensitivity, a high carrying capacity of multiple drugs, and enhanced penetration and retention in tumor tissues (37, 38). The research of nanomaterial-mediated sensitization of radiotherapy mainly focuses on the use of high atomic number nanoparticles (such as gold, silver, and bismuth) to enhance the radiation energy deposition in cells. With the development of polymer nanomaterials, the research on the treatment of glioma is increasing gradually. Small molecule drugs can be chemically bound and physically coated to target glioma tissues through the blood–brain barrier, thus improving the efficacy of radiotherapy for glioma.

### Nano-Radiotherapy Sensitizers Can Efficiently Cross the BBB and Target Gliomas

**Figure 1:**
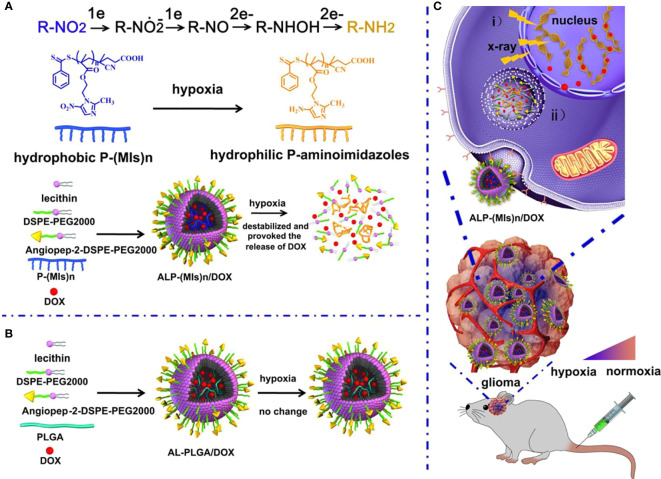
Schematic of the hypoxia-responsive and hypoxia RT sensitization ALP-(MIs)n drug-delivery system. **(A)** Mechanism of ALP-(MIs)n RT sensitization and DOX release under hypoxic condition and formation of ALP-(MIs)n/DOX. Six electrons are transferred in the complete reduction of nitro (R-NO_2_) to amine (R-NH_2_) under hypoxic conditions *via* a single-electron reduction catalyzed by a series of intracellular nitro reductases. **(B)** Formation of AL-PLGA/DOX as the control group. **(C)** Schematic illustrating ALP-(MIs)n applications: (i) Hypoxic cell radiosensitizer. ii. Hypoxia-responsive release of DOX into the cytoplasm, and then transports it to the nucleus to kill tumor cells (47).

The blood–brain barrier (BBB) is the outer layer of blood vessels in the brain and spinal cord, which is highly selective for substance penetration. The barrier properties of a healthy blood–brain barrier are mainly due to the tight junctions between endothelial cells, which are stable by astrocytes and pericytes. Through complex design, the blood–brain barrier can prevent the passage of neurotoxins and microorganisms, and selectively allow oxygen and nutrients to enter the central nervous system, thereby maintaining homeostasis (39–44). BBB restricts the delivery of chemical drugs and becomes a difficult point in the chemotherapy of glioma. Therefore, the primary problem that nano-radiosensitizers used in radiotherapy for glioma need to solve is to cross the BBB and target the glioma tissue. Normally, nanoparticles cannot pass through the BBB, but when the tumor is present, BBB permeability increases, and nanoparticles can pass through. Compared with normal tissues, tumor tissues have an abundant blood supply, wide vascular space, and lack lymphatic drainage, making macromolecular substances or lipid particles have high permeability and high retention effects in tumor tissues, which can be called the high permeability and retention effect (EPR) of solid tumors. It can increase the drug concentration in tumor tissue through the EPR effect, which is passive transport. Our research group (45) used this effect to design an RT-sensitive liposome that is responsive to hypoxia as a novel DOX delivery system. The hypoxia radiosensitizer nitroimidazole combines with lipid molecules with hydrolysable ester bonds to form MDH, which is mixed with DSPE-PEG2000 and cholesterol to make MLP liposomes. Experimental results show that MLP liposomes can carry DOX and nitroimidazole across the BBB and can effectively stay in the tumor area. Hypoxia can induce the conversion of hydrophobic nitroimidazole into hydrophilic aminoimidazole through electron transfer, causing the instability of liposomes and releasing DOX. Meanwhile, MI enhanced the radiosensitivity of radiation-tolerant hypoxic cells due to electron affinity, and DNA damage caused by ionizing radiation was enhanced. The drug delivery system can effectively inhibit the growth of C6 glioma cells by combining radiotherapy and chemotherapy. Additionally, nano-radiotherapy sensitizers actively cross BBB by adding special ligands, antiboding and proteining to the surface engineering of nanoparticles to form multifunctional nanoparticles, with a strong BBB crossing efficiency and can selectively and specifically target CNS tumor tissues (46). It should be noticed that there is another Nano-radiotherapy sensitizer that was designed by our group called ALP-(MIs)n/DOX, and it also has an excellent ability to cross the BBB (47) (**Figure 1**). Zhang et al. (48) encapsulated the cyclin-dependent kinase inhibitor dinaciclib into lipid nanoparticles containing anti-PD-L1 antibodies, and RT induced the up-regulation of PD-L1 in glioma infiltrating TAMC (Tumor-associated myeloid cells). Lipid nanoparticles (LNP) targeting PD-L1 effectively target glioma tissues, inhibit PD-L1 or eliminate TAMCs, which are immunosuppressive cells, strengthen anti-tumor immunity, and extend the survival time of mice.

### Enhance the Efficacy of Radiotherapy for Glioma Through Radiation Energy Deposition

**Figure 2:**
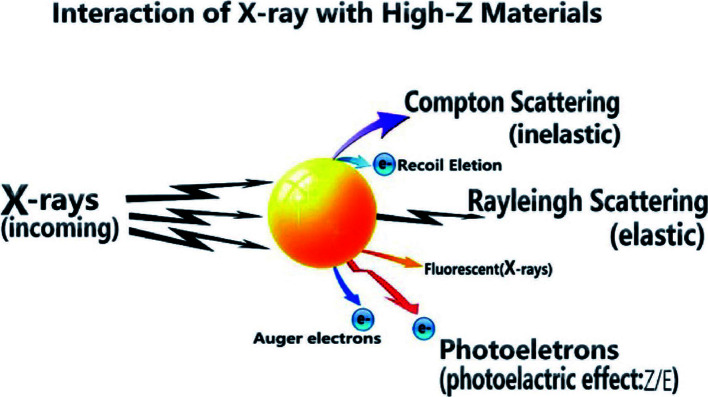
Radiant energy deposition to arouse secondary electrons (71).

In terms of sensitization of radiotherapy, metal nanoparticles have been studied for many years as radiotherapy sensitizers. Metal nanoparticles with a high Z value have a high absorption capacity of radiation and can concentrate radiation energy on the tumor site (49). It is generally believed that these nanoparticles increase the cross-section of tissues or cells that react with radiation, facilitating the efficient deposition of high-energy radiant energy. From the formula of X-ray absorption coefficient μ and incident X-ray energy E and atomic coefficient Z: μ = ρ Z^4^/(AE^3^), the absorption coefficient μ is positively related to the fourth power of atomic coefficient Z, where ρ is the density and A the atomic mass (50, 51). Therefore, materials with high atomic coefficient elements have better X-ray energy absorption. The high Z-value nanoparticles after absorbing ray energy can produce a photoelectric effect, Compton effect, and Auger effect; this then generates a series of secondary electrons, such as the photoelectron, Compton electron, and Auger electron (52–54), which can directly interact with biomolecules locally or generate large amounts of ROS with water molecules. The principium above is shown in **Figure 2** (71). Tumor cells are then killed and the sensitization of radiotherapy is enhanced. The radiosensitization effect of AuNPs depends on its size and the type of surface modification (55, 56). Silver, platinum, gadolinium, etc. have similar radiosensitization effects to gold nanomaterials. Liu et al. found that malignant glioma-bearing rats treated with silver nanoparticles (AgNPs) after radiotherapy effectively inhibited the proliferation of cancer cells and promoted the apoptosis of cancer cells (57).

### Enhance Radiotherapy for Glioma by Enhancing DNA Damage and Inhibiting DNA Repair

**Figure 3:**
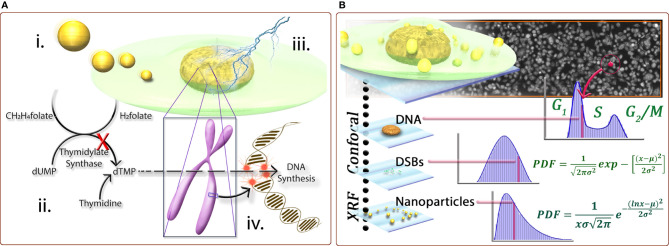
**(A)** Schematic representation of the following conceptst: (i) internalization of nanoparticles by cells can lead to the down-regulation of proteins, including thymidylate synthase (TS), important for DNA damage repair response; ii. due to the down-regulation of TS, the conversion of dUMP to dTMP is inhibited; iii. subsequently, when the DNA is subjected to insult by ionizing radiation causing doublestrand breaks; and iv. the normally effective homologous recombination pathway for repairing DSB’s in S-phase cells is also inhibited, leading to a biological mechanism of radiosensitization. **(B)** A cross-correlative methodology developed provides a three-dimensional data set to compare cell populations and sub-populations with regard to nanoparticle dose−response at the single-cell level. Correlating biological markers imaged with laser scanning confocal microscopy with elemental content from synchrotron X-ray fluorescence microscopy for cell populations provides statistically significant, descriptive analysis of cell populations with regard to biological response for a quantified number of nanoparticles. For example, only cells with comparable numbers of nanoparticles are compared, or only cells in a certain phase are compared. The population behavior can be described by fitting functions and any individual cell from a population can be characterized by its biological markers coupled with its nanoparticle content (67).

The radiotherapy resistance of tumors is mainly manifested in the double-strand breaks of tumor cells caused by radiation, and DNA itself has the ability to repair double-strand breaks (59). It is believed that the anti-radiation effect of tumors is due to hypoxia in tumor regions, which reduces DNA damage and enhances cellular defense mechanisms (60, 61). Therefore, DNA damage in glioma cells can be increased by increasing the oxygen content in the glioma region. In the meantime, the local oxygen of the tumor is more likely to produce ROS under the action of radiation, which increases the killing effect on the tumor. Many nano-radiotherapy sensitizers work by increasing the oxygen content of the tumor area (62, 63). Additionally, gliomas are usually resistant to RT due to their strong DNA repair activity (64, 65). The cytotoxicity of RT is mainly due to DNA damage, and double-strand breakage (DSB) caused by RT is the most serious type of DNA damage. If it is not repaired, it is deadly to the cells (66). Nanoparticles can inhibit DNA repair by inducing down-regulation of repair proteins, such as thymidylate synthase (67) (**Figure 3**), or inhibiting the DNA damage repair signaling pathway (68), thereby increasing the effect of radiotherapy. In terms of glioma, our research group designed a hypoxic radiosensitizer-prodrug liposome (MLP) as a carrier for the DNA repair inhibitor Dbait, which significantly inhibited the growth of glioma *in situ* in mice with the combination with radiotherapy (69).

### Can Effectively Transport Radionuclides to Achieve RIT

Radiotherapy is divided into two categories: external radiation therapy (EBRT) and internal radioisotope therapy (RIT). For EBRT, radiation beams such as high-energy X-rays, electron beams, or proton beams from outside the body are directly irradiated on the tumor, thereby inducing the death of cancer cells. For RIT, a minimally invasive method is used to introduce therapeutic radioisotopes into the tumor, such as direct infusion *via* a catheter (also called brachytherapy) (70, 71). Brachytherapy is not suitable for treating distant tumors due to the rapid elimination of radioisotopes *in vivo*. The combination of targeted nanoparticles with radioactive isotopes enables accurate isotope delivery, while nanoparticles for internal radiotherapy can also improve tumor vascular permeability, enhance retention effect (EPR), and increase uptake of the next wave of nanoparticles (38). In the treatment of glioma, nanoparticles were also widely used to deliver radionuclides (58, 72), which was proven to have good safety and feasibility (73). Allard introduced a lipid nanocapsule (LNC), which encapsulated ^188^Re(^188^Re(S_3_CPh)_2_(S_2_CPh)[^188^Re-SSS]) to form a lipophilic complex that can be used as a new type of radiopharmaceutical carrier. The results showed that the median survival of rats treated with 8Gy^188^Re-SSSLNC was significantly improved. Compared with the control group, the median survival time increased by about 80%, with 33% of long-term surviving animals and when administered in LNC,^188^Re tissue retention was greatly prolonged, with only 10% of the injected dose being eliminated at 72h (74). Interestingly, another study revealed that 188Re-activity gradient led to a bypass of immunosuppressive barriers, which can be used to treat glioblastoma (75).

### Nano-Radiotherapy Sensitizer Combined With Other Treatment Methods to Treat Glioma

Nano-radiotherapy sensitizers can not only be enriched at the tumor site by enhancing the penetration and retention effects and improving the targeting effect on tumor tissues, but they also can be combined with chemotherapy, immunotherapy, and other treatment methods. Meanwhile, the specific microenvironment of glioma is used to achieve effective drug delivery (76), improving the therapeutic effect of glioma.

**Figure 4:**
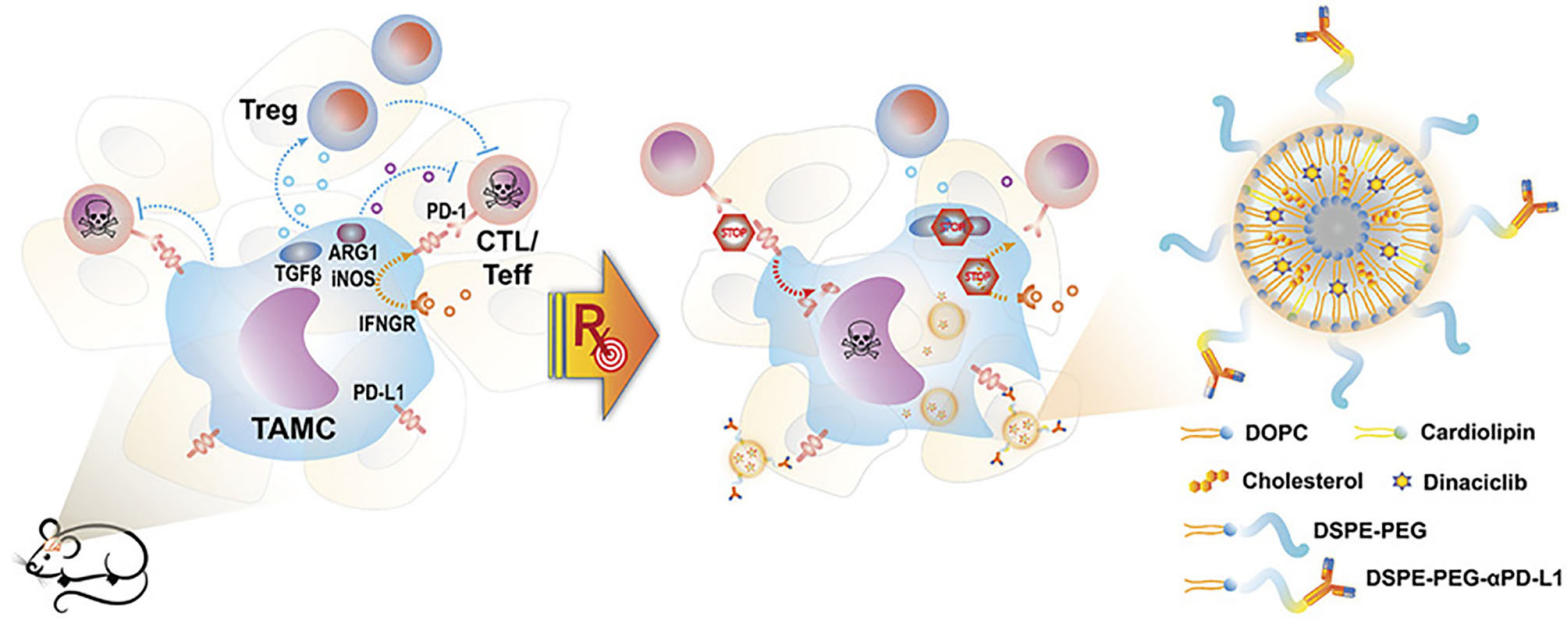
Schematic representation of nano-targeting of glioma-associated TAMCs. (CTL, cytotoxic T lymphocyte; Teff, effector T cell; PD-1, programmed cell death protein 1; IFNGR, IFN gamma receptor) (48).

Nano-radiotherapy sensitizer in combination with immunotherapy uses nano-delivery of inhibitory antibodies to block immune checkpoints. Due to the ability of nanomaterials to penetrate the BBB, immune-stimulating nanoradiation sensitizers can penetrate the BBB well and accumulate in glioma tissues. As mentioned above, lipid nanoparticles containing PD-L1 antibody not only have targeted functions but also inhibit PD-L1 and enhance T cell anti-tumor immunity and kill glioma cells in synergism with radiotherapy (48) (**Figure 4**). In addition, nanomaterials used as photosensitizers combined with photodynamic therapy (PDT) for radiotherapy have achieved significant effects on some other types of tumors (77, 78), which can also similarly kill glioma cells (79). In a study of high-grade glioma treatment, we found that photodynamic therapy (PDT) extended survival in patients, and in combination with intraoperative radiation therapy (IORT), improved survival even further (80). However, the application of nano-photosensitizer combined with PDT to the radiotherapy for glioma has not been reported in the literature.

Researchers found that enhanced autophagy of glioma promoter cells (GICs) contributes to the elimination of radiotherapy resistance (81). Liu et al. evaluated the radiosensitization effect of silver nanoparticles (AgNPs) on hypoxic glioma cells and found that the radiosensitization ability of AgNPs in hypoxic U251 cells and C6 cells was higher than that of normoxic U251 and C6 cells (82). The main reason for hypoxic radiation sensitization induced by siNPS is the promotion of cell apoptosis and the enhancement of destructive autophagy, suggesting that AgNPs can be used as excellent radiosensitizers in the treatment of hypoxic glioma. Paradoxically, earlier studies have found that gamma-ray-induced autophagy contributes to the radioresistance of these cells, and autophagy inhibitors may be employed to increase the sensitivity of GSCs to gamma-radiation (83).

Autophagy has a protective effect on inhibiting the radiosensitization of STAT3. Inhibition of autophagy and STAT3 may be a potential therapeutic strategy to improve the radiosensitization of glioma cells (84). Therefore, the effect of autophagy on radiosensitization of gliomas is still controversial (85, 86).

Emerging nano-radiosensitizers have developed rapidly currently. For example, near-infrared light combined with radiotherapy that converts light energy into heat energy (87), sonoporation sensitization radiotherapy (88), and nanoparticles of heterojunction structure can avoid the recombination of electrons and holes, improve photocurrent and photocatalytic activity, etc. (89).

## The Main Types of Nano-radiosensitizers in the Treatment of Gliomas

**Figure 5:**
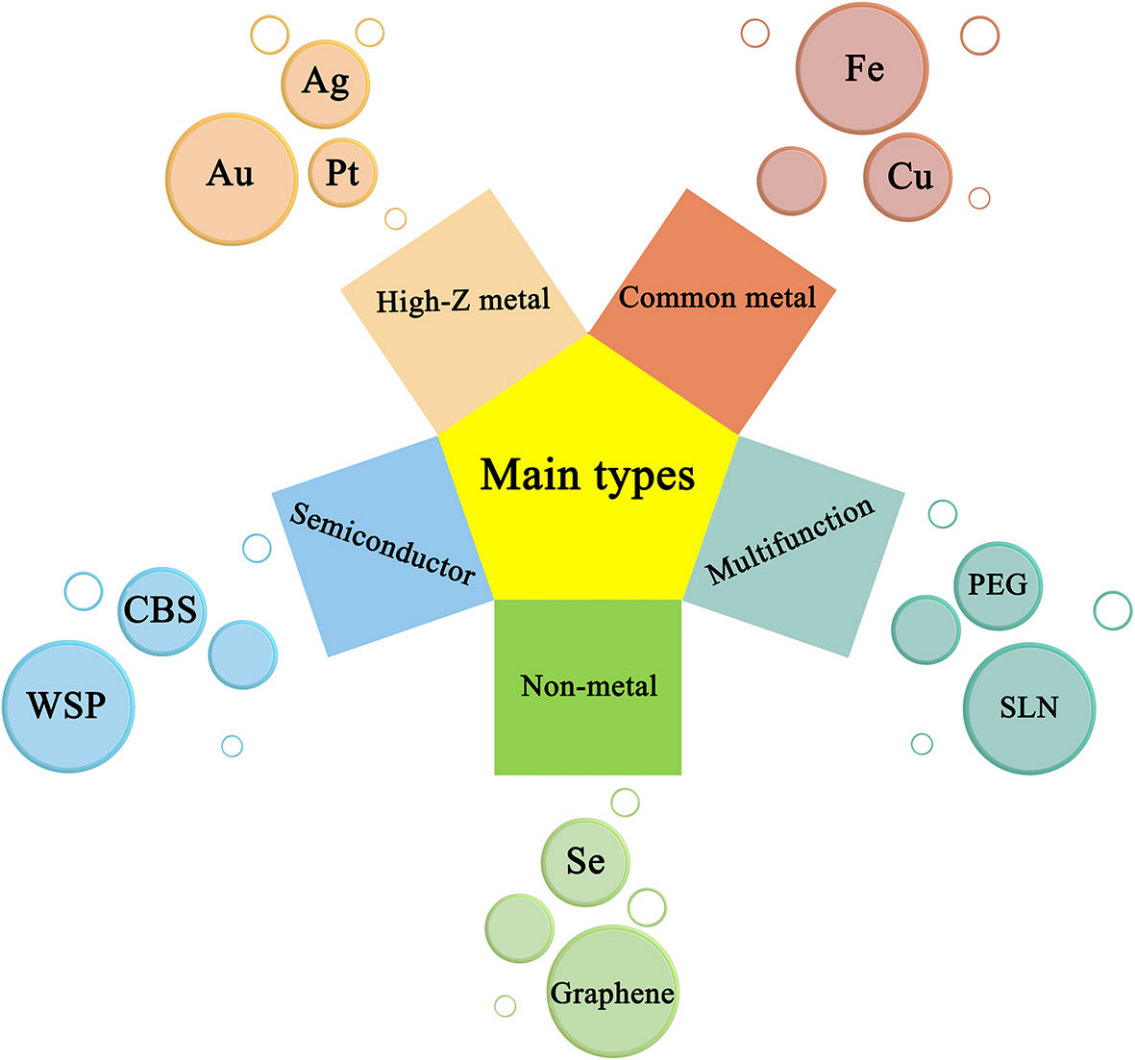
Representative nanomaterials and basic principles of action under types of nanoradiosensitizers.

Nano-radiotherapy sensitizers can overcome a series of problems such as high toxicity, non-specificity, and obvious side effects of traditional sensitizers, making nano-radiosensitization treatments become a popular treatment for various malignant tumors including gliomas. According to the physicochemical properties of nano-sensitizers in existing research, the common nano-sensitizers (nanoparticles) in the treatment of glioma are divided into the following categories: 1. High-Z metal nano-radiotherapy sensitizers; 2. Common metal and its oxide nano-radiotherapy sensitizer; 3. Semiconductor nano-radiotherapy sensitizer; 4. Non-metallic nano-radiotherapy sensitizer material; and 5. Multifunctional nano-radiotherapy sensitizer. We draw a diagram (**Figure 5**) which summarizes the main species of nano-radiosensitizers and more details are shown in **Table 1**.

**Table 1:** Lists the types of glioma nano-radiotherapy sensitizers mentioned in the paper, including the type, name, and position of sensitizers.

Main types	Based Nanomaterial	References
High-Z metal nano-radiosensitizers	Gold (Au)	(92–94, 96, 125)
	Silver (Ag)	(82)
	Platinum (Pt)	(96)
	gadolinium (Gd)	(98, 100, 126)
	Hafnium (Hf)	(99)
	Tantalum (Ta)	(97, 98, 101)
	Cerium (Ce)	(103)
	Terbium (Tb)	(104)
	Tungsten/Wolfram (W)	(105)
	Bismuth (Bi)	(106–110)
Common metal and its oxide nano-radiosensitizer	Iron (Fe)	(111, 112)
	Copper (Cu)	(113, 114)
	Fe_3_O_4_	(64)
	ZnFe_2_O_4_	(115)
Semiconductor nanomaterial sensitizer	WO_2.9_-WSe_2_-PEG (wsp)	(118)
	Cu_3_BiS_3_ (CBS)	(119)
Non-metallic nanomaterial sensitizer	Selenium (Se)	(107, 121)
	Graphene	(27, 122)
Multifunctional nano-radiotherapy sensitizer	SLN+EGFR+siRNA	(123)
	PEG+PEI+siRNA	(124)

### High-Z Metal Nano-Radiosensitizer

A high-Z metal nano-radiotherapy sensitizer is the most in-depth research among various nano-material sensitizers because high-Z elements have a strong X-ray attenuation ability (50), which can increase the radiation dose of tumor cells in GBM tissues, thereby achieving the therapeutic effect of sensitization of radiotherapy (90). Gold, silver, platinum, and other high-Z precious metals have the advantages of low toxicity, easy preparation, controllable size and morphology, easy surface functionalization, high chemical stability, and good biocompatibility (91), which have natural advantages of of preparing bio-related nanomaterials. Recently, gold nanomaterials, the most studied among high Z metals, have been widely used in radiosensitization therapy of glioma (92). Yan Liu et al. used a one-pot green syn-thetic method to synthesize luminescent gold nanoclusters (AuNC) (93). Su-Yang Yang et al. used the strategy of cross-linked stable lipid nanocapsules (NCs) as a carrier to prepare a kind of inter-membrane cross-linked multilayer lipid vesicle (ICMV) containing amphiphilic gold nanoparticles (amph-NPs) to form Au-NCs. In vivo experiments on mice showed that the AU-NCS combined radiotherapy group had an obvious tumor-killing effect compared with the radiotherapy alone group (94). Yijin Liu et al. studied a mixed anisotropic nanostructure composed of gold (Au) and titanium dioxide (TiO_2_). As a radiosensitizer, Au-TiO_2_ nanoparticles (DAT) can significantly enhance the effect of radiotherapy (77, 93). In addition to nano-gold, nano-silver and nano-platinum materials have also been extensively studied (95). Haiqian Zhang et al. prepared a silver nanoparticle (AgNPs) for radiosensitization of hypoxic glioma cells, with the results showing that AgNPs can significantly improve the effect of radiotherapy in the radiotherapy of hypoxic glioma (82). Eva Pagáˇ cová et al. analyzed effects on radiation-induced γH2AX+53BP1 lesions of different nanoparticle materials (platinum (Pt) and gold (Au)), cancer cell types (HeLa, U87, and SKBr_3), and low-line energy transfer (LET) ionizing radiation (γ- and X-rays) dose (up to 4Gy) to evaluate its radiosensitization effect in gliomas (96). In addition to the above high-Z precious metals, other high-Z metal nanosensitizers also include gadolinium (Gd), hafnium (Hf), tantalum (Ta), cerium (Ce), terbium (Tb), tungsten (W), bismuth (Bi), and other metal elements with large atomic coefficients (97). Particularly, lanthanide metal-based nanoparticles are being developed and utilized due to their strong X-ray attenuation ability. Verry, C et al. designed a gadolinium (Gd)-based AGuIX nanoparticle for combined radiotherapy for patients with brain metastases, showing that the nanoparticle significantly improved the effect of radiotherapy (98, 99). Chen has developed a nano-sensitizer of titanium dioxide doped with gadolinium, which targets mitochondria for effective radiation therapy. With X-ray irradiation, nanosensitizers trigger the domino effect of ROS accumulation in mitochondria (99). Géraldine et al. used 9L glioma cell line (9LGS) tumor-bearing mice to inject a biodegradable gadolinium-based ultrafine nanoparticle (AGuIX nanoparticles) intravenously. They found that AGuIX particles do not leak out of normal blood vessels, allowing more particles to accumulate effectively in glioma tissue, increasing the sensitivity of radiation therapy (100, 101).

In addition to the metal gadolinium (Gd), the metal hafnium (Hf), as a high-Z metal, is often used in the X-ray manufacturing industry because it easily emits electrons. Pure hafnium has the advantages of plasticity, easy processing, high temperature resistance, corrosion resistance, and so on. It is an important material in the atomic energy industry, which has also been put into medical research and use. Min-Hua Chen proposed a nanoparticle that can enhance active oxygen: Hf-doped hydroxyapatite (HF: HAP). After exposing (HF: HAP) to gamma rays, the generation of ROS in the cell increases significantly (99). Jin J summarized the latest progress in radiation therapy (RT) and immunotherapy of nanoparticles (NPs) such as hafnium (Hf) and bismuth (Bi) and evaluated the feasibility of high-Z metals as nano-radiosensitizers (102).

Among high-Z metals, tantalum (Ta) has been widely used in the medical field because of its moderate hardness and excellent ductility. The excellent corrosion resistance is mainly due to the formation of s atable tantalum pentoxide (Ta_2_O_5_) protective film on the surface, which has also been used in the field of radiotherapy for glioma sensitization. Briggs discovered for the first time that tantalum (Ta_2_O_5_) nanoparticles showed a dose-enhancing effect on gliosarcoma cells with strong radiation resistance under 10MV irradiation. It is believed that the enhancement effect is due to the secondary electrons generated by the photoelectric effect, which increases the biological effect of radiation, indicating that tantalum Ta_2_O_5_ has a certain radiosensitization effect in the radiotherapy for glioma (101). Besides, cerium (Ce) is also a widely used high-Z metal in the medical field as the most abundant rare earth element in the earth’s crust. Xiaoyan Zhong prepared Ce (Ce)-doped NaCeF_4_: Gd and Tb fluorescent nanoparticles (SCNP or fluorescent scintillator). Due to the sensitization of Ce ions, Tb ions can trigger X-ray sensitive fluorescence (XEF) under X-ray irradiation to generate reactive oxygen species (ROS) in RDT, thereby increasing the sensitivity to radiotherapy (103). Runowski enriches the fluorescence effect of CeF_3_ nanoparticles (NPs) by co-doping with Tb^3+^ and Gd^3+^ (CeF_3_: Gd^3+^, Tb^3+^) for the treatment of deep tumors such as intracranial tumors (104).

As a new high-tech material, tungsten (W) is another high-Z metal that has been put into the medical field. According to Wang, J’s research, tungsten sulfide (WS_2_QDs) is a nanomaterial suitable for radiotherapy (RT) and photothermal therapy (PTT), proving that tungsten (W) can be used as a nano-radiosensitizer (105).

Bismuth (Bi) is a hot spot nano-radiotherapy material besides nano-gold materials. Hossain, M controlled the concentration of nanoparticles to 350 mg·g−1 under a radiation source of 50 kVp and found that the radiosensitization effect of nano-bismuth was 1.25 times and 1.29 times stronger than that of nano-gold and nano-platinum, respectively. Based on this, it is concluded that bismuth nanoparticles have a stronger sensitizing effect than gold and platinum nanoparticles with the same nanometer size, particle concentration, and action site (106). In the presence of bovine serum albumin (BSA), Fangxin Mao et al. synthesized ultra-small biocompatible Bi_2_Se_3_ nanoparticles by reacting hydroxyethylthioselenide and bismuth chloride in an aqueous solution BSA-Bi_2_Se_3_ shows a strong wide absorption rate, high light-to-heat conversion efficiency, and a strong radiation sensitization effect in the near-infrared (NIR) window (107). Huan Yu et al. synthesized bismuth sulfide nanoparticles (BiNP) and coupled them with immunoactive Ganoderma lucidum polysaccharide (GLP) and verified that GLP-BiNP has a dual role in tumor treatment through radiosensitization and immune activity (108). Guosheng Song used a partial cation exchange method, which took MnSe nanocrystals as a template to replace manganese with bismuth in the outer layer to form a Bi_2_Se_3_ shell, to advance the blood supply of tumor tissue, increase oxygenation significantly, improve the effect of radiotherapy (RT), and kill tumor cells effectively (109). Fangmei Zhang et al. designed and prepared a multifunctional bismuth-based nano-olfactory, which was functionalized by S-nitrosothiol and named Bi-SNO (NPs). X-rays can break down the S-N bond and trigger the release of a large amount of NO (over 60μM). The prepared Bi-SNO (NPs) with a small volume (36 nm) has the ability to absorb and convert 808 nm near-infrared photons for photothermal treatment, as well as the ability to increase X-ray absorption and CT imaging sensitivity. Moreover, the synergistic effect of Bi-SNO radiation, photothermal, and gas therapy *in vivo* was further studied, to get a significant synergistic tumor inhibition effect (110).

### Common Metal and Its Oxide Nano-Radiosensitizer

Other common metal types with nanoradiosensitization effects include common non-high Z nanoradiosensitizers, such as nanoradiosensitizers, iron nanoradiosensitizers, and copper nanoradiosensitizers. Chengcheng Yang developed a polydopamine (PDA) coated Ge11 peptide conjugated iron oxide nanoparticles (Ge_11_-PDA-Pt@USPIOs) with cisplatin as a carrier, based on ultra-small superparamagnetic iron oxide nanoparticles (PAA@USPIos) coated with polyacrylic acid, showing synergistic therapeutic effects of radiotherapy and chemotherapy under low temperature *in vitro* (111). Muhammed prepared SiO-MNP-coated iron oxide nanoparticles by co-precipitation and other methods to enhance the radiation sensitization effect by increasing the production of ROS (112).

For nano-copper sensitizers, Yu Fan et al. designed a therapeutic nano-platform based on the complexation of pyridine (Pyr) functionalized fifth-generation (G_5_) polyamidoamine dendrimers with Cu^2+^, which is used for radio-enhanced T1-weighted magnetic resonance (MR) imaging and coordinated radiotherapy and chemotherapy for tumors and tumor metastases (113). Chenyang Zhang designed a new smart radiosensitizer based on Cu_2_(OH)Po_4_ nanocrystals. Sensitizers can respond to both endogenous (H_2_O_2_) and exogenous (X-rays) stimuli simultaneously and can finally induce apoptosis and necrosis of cancer cells (114).

Some ferrite-based spinel structure nano-material sensitizers have also been reported. For example, Alireza Meidanchi synthesized superparamagnetic zinc ferrite spinel nanoparticles ZnFe_2_O_4_ by a hydrothermal method which is used as a radiosensitizer for cancer treatment. When exposed to gamma rays, the low-energy electrons produced in the nanoparticles further kill tumor cells. The use of biocompatible ZnFe_2_O_4_ nanoparticles (at a concentration of 100μg/ml) in radiotherapy can produce a synergistic response to radiotherapy. The killing efficiency of highly radiation-resistant cancer cells is 17 times that of traditional radiotherapy, so it is a reliable radiation sensitizer (115). Besides, the sensitizers of metal nanomaterials for glioma include some special new nanometal materials, such as metal-organic skeleton (Zr-MOF) nanoparticles (116) and room temperature liquid nanometals (LMs) (117). Moreover, some of the above nano metal materials not only directly affect the sensitization of radiotherapy but also act as multifunctional adjuvants in auxiliary imaging, such as X-ray diagnosis (116).

### Semiconductor Nanomaterial Sensitizer

In the field of semiconductor nanosensitizer materials, common semiconductor materials include silicon (Si), germanium (Ge), gallium arsenide (GaAs), and other compound semiconductors doped or made into other compound semiconductor materials. Among them, silicon is the most commonly used semiconductor material. Semiconductors have the following in common. The conductivity of a semiconductor is between a conductor and an insulator, which will change significantly when it is stimulated by external light and heat. Therefore, semiconductor materials have great potential in the application of sensitization of radiotherapy. Dong Xinghua et al. discovered WO_2.9_-WSe_2_-PEG semiconductor heterojunction nanoparticles (WSP NPs), which can be combined with radiotherapy (RT), photothermal therapy (PTT), and immune checkpoint suppression therapy (CBT) to jointly enhance anti-tumor and anti-metastasis effects. Under X-ray irradiation, the nanosystem catalyzes the highly expressed H_2_O_2_ in TME, promotes the generation of non-oxygen-dependent reactive oxygen species, and enhances the effect of radiotherapy (118). Yiwei Kang et al. encapsulated small semiconductor copper bismuth sulfide (Cu_3_BiS_3_, CBS) nanoparticles and rare earth down-conversion (DC) nanoparticles in larger size zeolite imidazole skeleton-8 (ZIF8) nanoparticles and then loaded them with anticancer drugs Doxorubicin (DOX). Under X-ray irradiation, a moderate dose of CBS&DC-ZIF8@DOX composite material can achieve high (87.6%) tumor suppression efficiency and synergistic radiotherapy and chemotherapy (119).

### Non-Metallic Nanomaterial Sensitizer

The development of non-metallic nanomaterial sensitizers in the treatment of glioma has also been very rapid, such as selenium (Se) nanoparticles, graphene nanomaterials, etc. (120). Qian Huang et al. synthesized selenium nanoparticles by reducing tin dioxide with vitamin C. The selenium nanoparticles were used as sacrificial templates to react with copper ions to form copper selenide nanoparticles. The results showed that the dumbbell-like copper-gold selenide nanocrystals could be used as an effective radiosensitizer for enhanced radiotherapy (121).

In the treatment of gliomas, graphene nanomaterials have also made new progress in the field of sensitization and radiotherapy. Sakine Shirvalilou et al. used magnetic graphene oxide (NGO/SPIONs) nanoparticles (MNPs) coated with PLGA polymers as dynamic nanocarriers for IUDR to achieve 5-iodo-2 deoxyuridine (IUdR) entry into the blood–brain barrier (BBB). IUDR/MNPs were administered intravenously to tumor-bearing rats of the C6 glioma cell line under a magnetic field of 1.3T, and the synergistic effect of IUDR/MNPs and radiotherapy was found. Compared with radiation alone, increasing the ratio of Bax/Bcl-2 (2.13 times) can significantly inhibit tumor expansion (>100%) and prolong survival time (>100%). Inhibit the anti-apoptotic response of glioma rats, thereby enhancing the sensitizing effect of tumor radiotherapy (27). Lei Chen et al. developed ^131^I-labeled, polyethylene glycol (PEG) coated reduced graphene oxide (RGO) nanoparticle. After intravenous injection, gamma imaging shows a significant accumulation of ^131^IRGO-PEG in tumor tissue. Reduced graphene oxide has a strong near-infrared absorbance, which can effectively heat tumors under near-infrared irradiation. The ^131^I emits high-energy X-rays due to ionization, which induces tumor killing and enhances the effect of radiotherapy on cancer cells (122).

### Multifunctional Nano-Radiosensitizer

A simple nanoradiotherapy sensitizer cannot meet the needs of clinical treatment for the characteristics of radiation resistance and immunosuppression of glioma. Functional nanomaterials can improve the radiotherapy sensitivity of gliomas in many ways. Erel-Akbaba G has developed a cyclic peptide iRGD (CCRGDKGPDC)-conjugated solid lipid nanoparticle (SLN) to deliver epidermal growth factor receptor (EGFR) and PD-L1 small interfering RNA (SiRNA), binding to targeted and immunotherapy for glioblastoma and enhancing the efficacy of radiation therapy by regulating the immune system (123). Forrest M. Kievit et al. prepared a nanoparticle (NP) composed of superparamagnetic iron oxide core, biodegradable chitosan, polyethylene glycol (PEG), and polyethyleneimine (PEI) coating. The NP can bind to siRNA and protect it from degradation and deliver siRNA to the area around the target nucleus to use an siRNA vector to inhibit the expression of APE1 and enhance the sensitivity of brain malignancies to RT (124). siRNA itself is a radiotherapy sensitizer. By carrying a certain radiotherapy sensitizer nanocarrier and combining immunotherapy, it can achieve double or even multiple sensitizers, which is also the research focus of future radiotherapy sensitizer nanocarrier.

## Outlook

In summary, the combined application of nanoparticles and radiotherapy sensitizers can significantly improve the effect of radiotherapy. The special biological characteristics of glioma weaken the effect of traditional radiotherapy, and the excellent targeting and good biocompatibility of nano-radiosensitizers solve the difficulties of traditional radiotherapy for glioma. At present, nano-radiosensitizers have developed rapidly in the past few years, providing new research strategies for sensitization of radiotherapy and new ideas for radiotherapy for gliomas. As mentioned earlier, nanoparticles as radiosensitizers have shown great potential in tumor treatment. New drug delivery methods can also improve the sensitizing effect of radiosensitizers (127). Nano-radiosensitizers are characterized by low cytotoxicity, good targeting, good biocompatibility, and easy functionalization. They can pass the blood–brain barrier (BBB), and some of them have been used as radiosensitizers in clinical treatment (128). However, single-functional nanoparticles cannot fully meet clinical needs, and more and more researchers have focused on finding multifunctional nanoparticles that are more conducive to clinical transformation. Furthermore, improving the drug-carrying capacity of nanomaterials is a strategy to develop multifunctional platforms. Research on the radiation sensitization mechanism will provide targets for new radiation sensitizers, and interdisciplinary research will promote the further development of new radiation sensitizers (129).

## Author Contributions

HL and YX was responsible for the overall idea of the article. YH was responsible for the abstract and the fourth part. XZ was responsible for the *Introduction* and *Outlook* and revised the format. HM was responsible for the writing of the second part. LL was responsible for the writing of the third part. All authors contributed to the article and approved the submitted version.

## Funding

This work was supported by grants from the National Natural Science Foundation of China [No. 81772665], Social Development Project of Jiangsu Department of Science and Technology [No. BE2020647, BE2020642], Jiangsu provincial Commission of Health and Family Planning [No. Q201608], Six Talents Peak Foundation of Jiangsu Province [No. 2018-WSW-071], the Youth Science and Technology Innovation Team of Xuzhou Medical University [No. TD202002], and the Social Development Project of Xuzhou Department of Science and Technology [No. KC20079].

## Conflict of Interest

The authors declare that the research was conducted in the absence of any commercial or financial relationships that could be construed as a potential conflict of interest.
